# Assessment of the Feasibility and Cost of Hepatitis C Elimination in Pakistan

**DOI:** 10.1001/jamanetworkopen.2019.3613

**Published:** 2019-05-10

**Authors:** Jagpreet Chhatwal, Qiushi Chen, Xiaojie Wang, Turgay Ayer, Yueran Zhuo, Naveed Z. Janjua, Fasiha Kanwal

**Affiliations:** 1 Harvard Medical School, Boston, Massachusetts; 2 Massachusetts General Hospital Institute for Technology Assessment, Boston; 3 Harold and Inge Marcus Department of Industrial and Manufacturing Engineering, Pennsylvania State University, University Park; 4 Department of Industrial and Systems Engineering, University of Florida, Gainesville; 5 H. Milton Stewart School of Industrial and Systems Engineering, Georgia Institute of Technology, Atlanta; 6 British Columbia Centre for Disease Control, Vancouver, British Columbia, Canada; 7 School of Population and Public Health, University of British Columbia, Vancouver, British Columbia, Canada; 8 Houston Veterans Affairs Health Services Research and Development Center of Excellence, Michael E. DeBakey Veterans Affairs Medical Center, Houston, Texas; 9 Department of Medicine, Gastroenterology and Hepatology, Baylor College of Medicine, Houston, Texas

## Abstract

**Importance:**

Chronic hepatitis C virus (HCV) infection is a global health problem. The World Health Assembly recently pledged to eliminate HCV by 2030. However, in Pakistan, a country with one of the highest prevalence rates, the feasibility and cost of HCV elimination are not known.

**Objectives:**

To investigate whether and under what conditions HCV elimination is feasible in Pakistan and to estimate the cost of such elimination.

**Design, Setting, and Participants:**

This decision analytical model study used a microsimulation model of the HCV epidemic in Pakistan from 2015 to 2030. Using Pakistan-specific variables, the model simulated the landscape of HCV in Pakistan and evaluated the minimum required screening and treatment rates needed to eliminate HCV in Pakistan. The study used simulated individuals chronically infected with HCV from 2015 to 2030. The analysis was performed in 2018.

**Interventions:**

The status quo and 7 scenarios that can lead to HCV elimination in Pakistan by 2030, which were defined by different combinations of tests for screening, detection of viremia before treatment, and confirmation of treatment response.

**Main Outcomes and Measures:**

Temporal trends in HCV infection prevalence, mortality, and disability-adjusted life-years and total cost of HCV infection care under the status quo and scenarios that can eliminate HCV by 2030.

**Results:**

Under the status quo, from 2015 to 2030, 1.44 million people are projected to die of HCV infection; 48% of deaths would be among people younger than 50 years. To achieve HCV elimination in Pakistan, HCV testing would need to be scaled up to at least 25 million people to diagnose 900 000 persons and treatment to 700 000 people per year. Compared with the status quo, the elimination scenario would avert 323 000 liver-related deaths and 13.0 million HCV-associated disability-adjusted life-years from 2015 to 2030. The elimination scenario was associated with cost savings of $2.6 billion from 2018 to 2030 with use of a point-of-care test for population-wide antibody screening and detection of viremia and treatment response.

**Conclusions and Relevance:**

Substantial scale-up of HCV testing and treatment may be essential to eliminate HCV infection in Pakistan, and such a strategy may be associated with cost savings in the near future. Although HCV elimination in Pakistan may be ambitious, strategic planning and strong support from the government may aid in its elimination.

## Key Points

**Question:**

Is hepatitis C virus elimination feasible in Pakistan, and what is the cost?

**Findings:**

This decision analytical model study found that an estimated 25 million people would need to be screened every year to diagnose 900 000 hepatitis C virus infections and 700 000 patients would need treatment per year to eliminate hepatitis C virus infection in Pakistan. This strategy was estimated to be associated with 323 000 liver-related deaths averted and cost savings of $2.6 billion from 2015 to 2030.

**Meaning:**

Substantial scale-up of hepatitis C virus testing and treatment may be essential to eliminate hepatitis C virus infection in Pakistan, and such a strategy may be associated with cost savings in the near future.

## Teaser

This decision analytical model study evaluates whether hepatitis C virus elimination is feasible in Pakistan and estimates the costs of eliminating this virus.

## Introduction

Chronic hepatitis C virus (HCV) infection is a global health problem that affects 70 million people worldwide.^1^ It is associated with a substantial morbidity and mortality burden, and in 2015, approximately 400 000 people died of HCV infection. Pakistan has a generalized epidemic of HCV infection and one of the highest prevalence rates (4.9%) in the world, with 8 million to 11 million individuals with active HCV viremia.^1,2^ Furthermore, most HCV-infected individuals in Pakistan remain undiagnosed.^2^ Most HCV infections in Pakistan are transmitted through unsafe medical practices, especially reuse of injection equipment in the health care setting.^3^ In addition, people who inject drugs, although a small proportion of the population, have a high prevalence of infection, ranging from 40% to 90%.^4,5^

The recent availability of new direct-acting antivirals (DAAs) offers an opportunity to eliminate HCV. The World Health Assembly pledged to eliminate HCV as a public health threat (90% reduction in HCV infection incidence and 65% reduction in HCV infection mortality) by 2030. To achieve this elimination goal, 90% of HCV-infected people need to be diagnosed and 80% of eligible people need to be treated by 2030.^6^

Despite the availability of DAAs, their high price in most high- and middle-income countries has kept the treatment uptake low.^7^ However, in Pakistan, generic DAAs are available at a price as low as $60 per treatment course, which represents one of the lowest prices worldwide. However, lack of a systematic screening program could hinder elimination efforts. The Pakistan government recently launched its first National Hepatitis Strategic Framework, which closely follows the World Health Organization (WHO) global health sector strategy on viral hepatitis while accommodating for Pakistan’s limited resources.^2,8^ However, critical gaps remain in identifying and testing HCV-infected persons and linking them to treatment. Currently, no formal recommendations on population-based screening for HCV in Pakistan have been made. In the absence of any screening program, the goal to eliminate HCV by 2030 may remain unrealized.^9^

Furthermore, although the cost of treatment is low, the cost of diagnostic testing remains substantial and is higher than the treatment cost in Pakistan. A large initial investment is likely needed to scale up HCV testing and treatment to millions of HCV-infected individuals in Pakistan. The amount of budget allocation and potential cost savings from these investments and the association with different HCV testing algorithms for eliminating HCV are not known.^1,10,11^ Our objective was to investigate whether and under what conditions HCV elimination is feasible in Pakistan, a country with one of the highest burdens of HCV infection, and to estimate the cost of HCV elimination.

## Methods

### Overview

We adapted a previously developed mathematical model, Hepatitis C Disease Burden Simulation (HEP-SIM), to simulate the landscape of HCV in Pakistan. The HEP-SIM model is an individual-level state transition model that simulates the clinical management of HCV by incorporating HCV natural history, different treatment waves, diagnosis rate, multiple testing algorithms, and access to antiviral therapies (eFigure 1 in the Supplement) from 2015 to 2030.^12,13,14^ We adapted the HEP-SIM model using Pakistan-specific data and used the model to project the disease and cost burden under the status quo and HCV elimination scenario using different HCV testing algorithms. The analysis was performed in 2018. All data used in this study were publicly available and therefore did not require approval from an institutional review board. We followed the Consolidated Health Economic Evaluation Reporting Standards (CHEERS) reporting guideline.^15^ We describe the major model components of the HEP-SIM model; further model details can be found elsewhere.^12,13,14^

### Patient Demographics

The base-case population in the HEP-SIM model represented HCV-infected patients in Pakistan. In 2008, the prevalence of chronic HCV infection was 4.9% (ie, 8.3 million people).^16^ The HCV genotype, age and sex distributions, chronic stages of HCV infection, and antiviral treatment history are detailed in eTable 1 and eTable 2 in the Supplement.

### Natural History of HCV Infection

The natural history of HCV infection in the HEP-SIM model was defined using acute and chronic phases of HCV infection, with chronic HCV infection defined using METAVIR fibrosis scores (F0, no fibrosis; F1, portal fibrosis without septa; F3, numerous septa without fibrosis; and F4, cirrhosis), decompensated cirrhosis, hepatocellular carcinoma, liver transplant, and liver-related death (eFigure 2 in the Supplement). We used a published meta-analysis^17^ to estimate fibrosis progression from F0 to F4 (eTable 3 in the Supplement). We estimated disease progression in HCV sequelae from published observational studies.^18,19,20^ Because few liver transplants occur in Pakistan, we assumed that liver transplant was not a viable option for patients with HCV infection.

We calibrated the annual incidence of HCV infection in Pakistan to 280 000 cases in 2014 and assumed it changes proportional to the change in the prevalence of HCV infection over time. We further conducted a sensitivity analysis considering that the incidence of HCV infection remains constant at 280 000 cases per year^1,21^ and increases at an annual rate of 2%.^10^

### Awareness and Screening for HCV Infection

We assumed that 95% of HCV-infected patients in Pakistan were not aware of their infection status in 2001.^22,23^ Undiagnosed patients could become aware of their HCV status by sporadic testing or through a screening program. We implemented sporadic HCV testing until 2017, reflecting the current practice in Pakistan. We assumed that among those who were offered testing, 91% would accept it and 90% of those who tested positive would receive those results.^13,24^ From 2018 onward, we simulated 2 scenarios for HCV screening: (1) status quo and (2) HCV elimination screening program, which was defined as 1-time HCV testing of all adults in Pakistan. Using our model, we identified the minimum annual diagnosis rate and the treatment rate needed to achieve HCV elimination by 2030, as defined by the WHO.

### HCV Testing Algorithms

Under the status quo, we simulated screening based on tests currently used in Pakistan: antibody test for screening, nucleic acid test (NAT) for detection of viremia, and assessment of treatment response. For the HCV elimination scenario, we simulated 7 testing algorithms for diagnosis of HCV infection.^25,26^ These included different combinations of tests for screening, detection of viremia before treatment, and confirmation of sustained virologic response (SVR) status after treatment (Table). The choice of these testing algorithms was based on the WHO testing guidance for middle- and low-income countries as well as the strategies currently considered in Pakistan.

**Table.** Different Hepatitis C Virus Testing Algorithms Evaluated to Scale Up Interventions in Pakistan^a^

Testing Algorithm	Screening Test	Detection of Viremia	Assessment of Hepatitis C Treatment Response
T1 (base case)	Laboratory based	Nucleic acid test	Nucleic acid test
T2	Laboratory based	HCVcAg test	Nucleic acid test
T3	Point of care	Nucleic acid test	Nucleic acid test
T4	Point of care	GeneXpert	GeneXpert
T5	Point of care	HCVcAg test and nucleic acid test if HCVcAg test result is negative	HCVcAg test
T6	Point of care	HCVcAg test and nucleic acid test if HCVcAg test result is negative	Nucleic acid test
T7	Laboratory based	GeneXpert	GeneXpert

Abbreviation: HCVcAg, hepatitis C virus core antigen.

^a^ Performance characteristics of each test are as follows: point-of-care screening, 99.5% sensitivity and 99.8% specificity; nucleic acid test, 99.8% sensitivity and 99.7% specificity; GeneXpert, 99.8% sensitivity and 99.7% specificity; HCVcAg test, 93.2% sensitivity and 98% specificity; and HCVcAg test and nucleic acid test if HCVcAg test result is negative, 99.8% sensitivity and 99.8% specificity.

### HCV Treatment Waves

We modeled antiviral treatment in different waves that reflected clinical practice in Pakistan: pegylated interferon and ribavirin until 2015, followed by the availability of oral DAA combinations from 2016 onward. We differentiated treatment regimens by treatment naive and experienced, interferon tolerance status, and presence of cirrhosis, as described in a previous study.^12^ The SVR rates of each type of treatment were estimated from real-world data and were based on HCV genotype, fibrosis stage, and treatment history (eTable 4 in the Supplement). We further assumed that patients who did not achieve SVR could receive treatment again 2 more times at most.

### Treatment Uptake Rate

Because all patients with a diagnosis cannot be treated in a single year, we implemented an annual treatment uptake rate defined by the number of patients who can receive HCV treatment each year. We assigned priority to patients with F3 and F4 scores for HCV treatment, followed by patients with F0 to F2 scores. We used historic data on the number of patients receiving antiviral treatment in Pakistan (eTable 5 in the Supplement). Under the status quo, we assumed that the annual treatment uptake rate would have stayed at the current rate of 160 650 per year from 2018 onward. In the HCV elimination scenario, we determined the number needed to treat from 2018 onward to achieve HCV elimination.

### Costs

To evaluate the cost of HCV elimination, we incorporated the following 3 cost components from a payer’s perspective: cost of HCV testing, cost of antiviral treatment,^27^ and cost of management of HCV-associated health states (eAppendix and eTable 6 in the Supplement) To estimate the cost of HCV testing, we calculated the number of people needed to screen to diagnose 1 HCV infection and multiplied that number by the cost of testing antibody and viremia. We considered 7 different testing algorithms using different tests and accounted for differences in their performance characteristics and costs (Table and eTable 6 in the Supplement).^25,26^ We also accounted for the programmatic cost of delivering the test. For DAA treatment, we used the price of $60 per treatment course^27^ and added the cost of testing for assessing treatment response.^27^ We estimated HCV-associated health state costs (scores of F0-F4, cirrhosis, and hepatocellular carcinoma) using the WHO Choosing Interventions That Are Cost-effective (CHOICE) project (eTable 6 in the Supplement).

### Model Outcomes

We projected temporal trends in HCV-associated disease burden in Pakistan under the status quo and HCV elimination scenario, which was defined as the minimum required annual HCV screening and treatment rate that would result in HCV elimination in Pakistan by 2030. To determine WHO’s target of achieving a 90% diagnosis rate, we defined the diagnosis rate as the percentage of diagnosed viremic cases and cured cases among the total population with HCV infection (ie, all uncured and cured cases) and defined treatment coverage (80% target) as the percentage of cured cases among the total population with HCV infection. We also estimated disability-adjusted life-years (DALYs) averted from scaling up HCV testing and treatment. To calculate DALYs, we used disability weights defined by the Global Burden of Disease study^28^ (0 for scores of F0–F4, 0.194 for decompensated cirrhosis, and 0.508 for hepatocellular carcinoma). We also conducted probabilistic sensitivity analysis by accounting for uncertainty in all model variables and presented results using 95% credible intervals. We estimated the cost of HCV management under the status quo and HCV elimination scenario for each of the 7 testing algorithms.

## Results

### Feasibility of HCV Elimination in Pakistan

**Figure 1.**
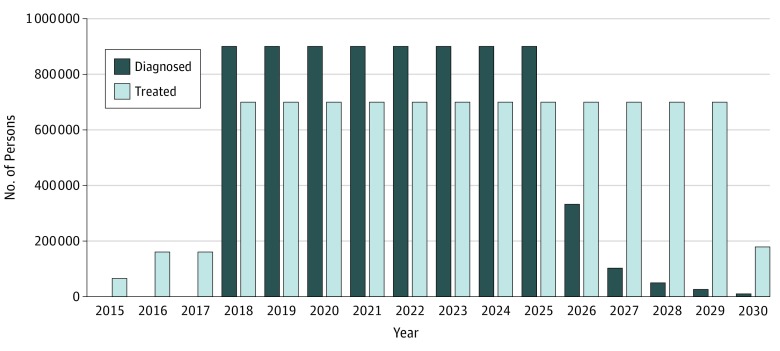
Number of Persons Who Need to Be Diagnosed and Treated in Pakistan Each Year to Meet the World Health Organization Target of Hepatitis C Virus Elimination The annual diagnosis rate would need to be scaled up to at least 900 000 cases per year, and the annual treatment rate would need to be scaled up to at least 700 000 persons per year to eliminate hepatitis C virus by 2030.

To achieve the WHO’s 2030 targets of HCV elimination in Pakistan, our model estimated that HCV testing would need to be scaled up to at least 25 million people to diagnose at least 900 000 cases per year from 2018 onward (Figure 1). Assuming the seroprevalence of 4.9%, these estimates translate into screening 24.9 million individuals per year in the general population. In addition, the treatment rate would need to be scaled up to at least 700 000 persons per year.

### HCV Disease Burden: Status Quo vs Elimination Scenario

**Figure 2.**
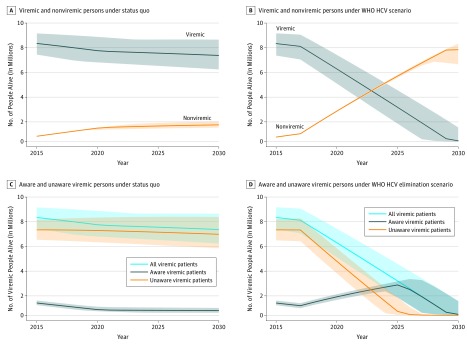
Number of Viremic (Aware and Unaware) and Cured Individuals in Pakistan From 2015 to 2030 Under Status Quo vs World Health Organization (WHO) Hepatitis C Virus (HCV) Elimination Scenario The elimination scenario was defined as an annual diagnosis rate of at least 900 000 cases per year and an annual treatment rate of at least 700 000 persons per year. Under the elimination scenario, the number of cured patients would exceed the number of viremic patients in year 2023. Bands show 95% uncertainty intervals generated by probabilistic sensitivity analysis.

In 2015, the number of patients with viremia living in Pakistan was estimated at 8.27 million (95% uncertainty interval [UI], 7.33 million to 9.12 million). Under the status quo, the number of patients would decrease modestly to 7.25 million (95% UI, 6.07 million to 8.59 million) by 2030 (12% reduction compared with 2015) (Figure 2A). During the same period, the number of cured patients is projected to increase from 0.41 million (95% UI, 0.39 million to 0.42 million) in 2015 to 1.37 million (95% UI, 1.14 million to 1.66 million) in 2030 (234% increase). In contrast, under the HCV elimination scenario, the number of patients with viremia would decrease to 89 200 (95% UI, 29 800-1 211 204) by 2030 (99% reduction compared with 2015) (Figure 2B), and the number of cured patients would increase to 7.76 million (95% UI, 6.53 million to 8.27 million) in 2030 (1790% increase compared with 2015).

In 2015, a total of 1.04 million (95% UI, 0.87 million to 1.25 million) individuals were aware of their HCV infection (ie, a diagnosis rate of 12.0%). Under the status quo, the diagnosis rate by 2030 would moderately increase to 16.8% (Figure 2C). In contrast, under the HCV elimination scenario, the diagnosis rate would increase to 99.9% by 2030 (Figure 2D).

**Figure 3.**
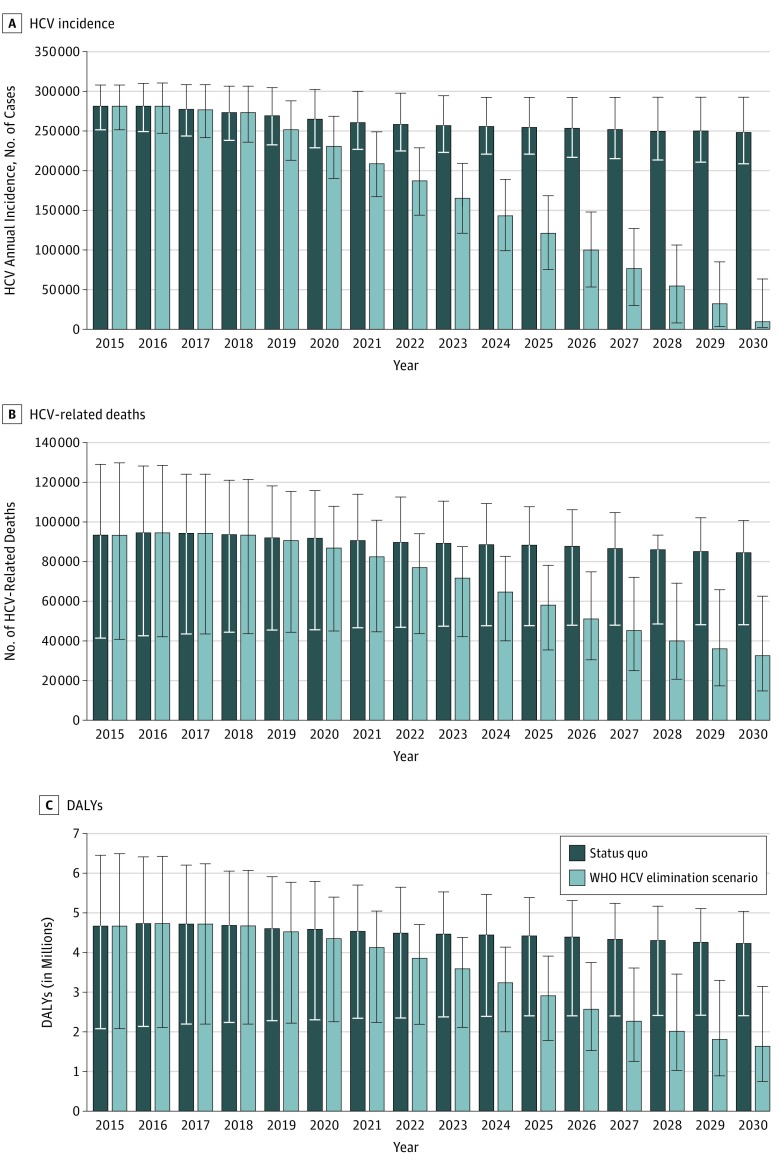
Annual Incidence of Hepatitis C Virus (HCV) Infection, Number of HCV-Associated Deaths, and Disability-Adjusted Life-Years (DALYs) in Pakistan Under Status Quo and World Health Organization (WHO) HCV Elimination Scenario From 2015 to 2030 The elimination scenario was defined as annual diagnosis rate of at least 900 000 cases per year and annual treatment rate of at least 700 000 persons per year. Error bars indicate 95% uncertainty intervals.

We also projected the estimated incidence of HCV infection over time (Figure 3A). Under the status quo, the incidence of HCV infection would decrease modestly from 281 000 cases per year (95% UI, 250 800-307 900) in 2015 to 248 000 cases per year (95% UI, 208 500-292 400) in 2030 (12% decrease). In contrast, scaling up HCV diagnosis and treatment (HCV elimination scenario) would substantially reduce HCV incidence to 9200 (95% UI, 1600-63 100) by 2030 (97% decrease compared with 2015).

We estimated that in 2015, a total of 93 300 (95% UI, 41 400-129 100) people died of HCV infection in Pakistan. Under the status quo, the annual number of HCV-related deaths would remain relatively unchanged (Figure 3B). From 2015 to 2030, an estimated 1.44 million people (1.15 million from 2018 to 2030) are projected to die of HCV infection in Pakistan; 48% of HCV-related deaths would occur in people younger than 50 years (eFigure 3 in the Supplement). In 2015, HCV infection was associated with 4.38 million (95% UI, 1.95 million to 6.05 million) DALYs (Figure 3C). Under the status quo, cumulatively, 60.74 million DALYs were associated with HCV infection between 2015 and 2030. The HCV elimination scenario would decrease HCV-associated deaths to 32 600 (95% UI, 14 800-62 800) by 2030 (65% reduction compared with the 2015 value). This strategy would avert 323 000 liver-related deaths and 13.0 million HCV-associated DALYs from 2015 to 2030.

We also projected that the cumulative incidence of decompensated cirrhosis from 2015 to 2030 would decrease from 1.10 million under the status quo to 0.62 million (44% reduction) under the HCV elimination scenario. Similarly, the cumulative incidence of hepatocellular carcinoma from 2015 to 2030 would decrease from 774 000 under the status quo to 547 000 new hepatocellular carcinoma cases (29% reduction) under the HCV elimination scenario.

### Cost of HCV Elimination

**Figure 4.**
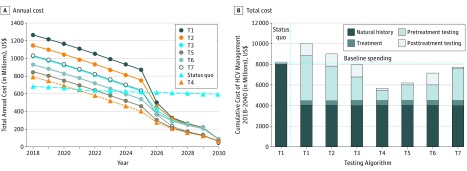
Cost of Hepatitis C Virus (HCV) Management From 2018 to 2030 Under Status Quo vs World Health Organization (WHO) HCV Elimination Scenario The elimination scenario was defined as annual diagnosis rate of at least 900 000 cases per year and annual treatment rate of at least 700 000 persons per year. A, Comparison of annual cost of HCV management under status quo vs WHO elimination scenario using different testing algorithms (as defined in the Table). B, Total cost of HCV management from 2018 to 2030 under status quo and different testing algorithms that result in HCV elimination by 2030. Testing algorithms T3 through T7 were associated with cost savings compared with the cost under status quo. The T4 algorithm provided the lowest cost of HCV management ($5.6 billion) and was associated with $2.6 billion in cost savings compared with the cost of status quo ($8.2 billion) (triangles).

We estimated that the total cost of HCV management in 2018 was $684 million. Under the status quo, the annual cost of HCV management would decrease modestly to $597 million by 2030 (Figure 4A); this projected decrease in costs was associated with deaths from HCV infection. Under the HCV elimination scenario using currently recommended diagnostics (antibody for HCV screening followed by nucleic acid testing for viremia diagnosis and assessment of treatment response [T1 algorithm]), the cost of HCV management would initially increase to $1.26 billion in 2018 but would decrease substantially to $89 million by 2030. Testing algorithm T4 (point-of-care test for HCV screening, GeneXpert for detection of viremia, and GeneXpert for the assessment of treatment response) yielded the lowest annual cost of $791 million in 2018, which is projected to decrease to $64 million in 2030. The annual cost under this scenario would decrease below the annual cost under the status quo in 2021 (ie, 3 years after scaling up screening and treatment).

Figure 4B shows the total costs of HCV management under the status quo and HCV elimination scenario using 7 HCV testing algorithms. Under the status quo, the total cost of HCV management from 2018 to 2030 would be $8.2 billion: $0.22 billion (2.7%) would be spent on HCV testing, $0.06 billion (0.7%) on antiviral treatment, and $7.93 billion (96.6%) on the management of HCV sequelae. With use of the currently recommended diagnostic tests (T1), the total cost of HCV management would be $10.0 billion: $5.54 billion (55.3%) would be spent on HCV testing, $0.51 billion (5.1%) on antiviral treatment, and $3.96 billion (39.6%) on management of sequelae. Compared with the status quo, the HCV elimination strategy would cost an additional $1.8 billion from 2018 to 2030. In contrast, testing algorithms T3 to T7 would result in cost savings compared with the cost incurred under the status quo. The T4 testing algorithm yielded the lowest total cost of HCV management of $5.6 billion from 2018 to 2030, which would result in cost savings of $2.6 billion compared with the status quo.

### Scenario Analysis

We evaluated model outcomes under 3 scenarios: (1) incidence of HCV infection increased by 2% per year, (2) HCV infection prevalence in 2018 was 20% higher (ie, 9.6 million instead of 8.0 million [base case]), and (3) the HCV infection awareness rate in 2018 was 7% instead of 12.7% (base case). We found that the annual diagnosis and treatment rates needed to eliminate HCV remained within 5% to 30% of the values reported in the base case (eTable 7 in the Supplement). In addition, the number of deaths averted, DALYs averted, total cost of HCV elimination, and cost savings associated with HCV elimination remained within 20% of the values estimated in the base case. Our results were sensitive to the HCV incidence scenario: HCV elimination would not be feasible if the incidence does not decrease over time.

## Discussion

Our study projected the trajectory of HCV infection in Pakistan. On the basis of current HCV management practices, the HCV burden ould continue to remain substantial in Pakistan. We projected that from 2015 to 2030, a total of 1.44 million people could die of HCV infection and that most patients with HCV infection would die young. Scaling up HCV testing and treatment could avert 323 000 liver-related deaths and as many as 13.0 million HCV-associated DALYs from 2015 to 2030, ultimately resulting in HCV elimination by 2030. Furthermore, scaling up HCV testing using innovative diagnostics could save $2.6 billion during the same period.

We found that HCV elimination is feasible in Pakistan; however, substantial efforts are needed—at least 25 million people would need to be screened every year to diagnose 900 000 HCV infections, and at least 700 000 patients would need treatment per year. This capacity target, representing a 4-fold increase in treatment and 24-fold increase in diagnosis compared with the current rates, may serve as important guideposts to determine progress toward achieving the overall goal of HCV elimination by 2030. Our results emphasize that HCV elimination in Pakistan may not be feasible without meeting these intermediate capacity targets.

Our results underscore the urgency to accelerate the diagnosis of HCV infection and identify the millions of individuals with undiagnosed HCV infection. Although HCV testing and treatment scale-up are happening in Pakistan, treatment uptake is happening at a faster rate than testing. Several HCV microelimination projects are under way, and these projects play an important role in identifying infected individuals and linking them to treatment. Elimination of HCV in Pakistan will require screening of the general population because of the diffuse nature of the country’s epidemic of HCV infection; this is challenging but could be optimized through first targeting subpopulations with higher prevalence and improving their linkage to treatment after diagnosis. The lessons learned from these initial efforts can then be extended to other subgroups with intermediate prevalence.

Increasing the diagnosis and treatment capacity would also require an initial investment, but our results show that such a strategy could be cost saving in the long term, especially if innovative approaches are used for confirming the diagnosis. In particular, we found that using a point-of-care test for antibody screening and HCV core antigen/GeneXpert for confirmation of SVR may reduce the overall cost of HCV elimination by 21% compared with a strategy that relies on current diagnostics. If the cost of new diagnostics could be further reduced, the amount of cost savings could be greater than $2.6 billion.

Although HCV elimination in Pakistan may be ambitious, we believe that strong support from the government and strategic planning can make elimination a reality. The foundation of scaling up testing and treatment has already been set up through Pakistan’s Hepatitis Prevention and Treatment Program.^29^ Efforts could focus on establishing a national policy for screening the general population for HCV infection, setting screening and testing targets for local health facilities, skill building of health care workers to expand access to screening and treatment, increasing availability of low-cost diagnostics and treatment, simplifying of diagnosis and treatment algorithms, and expanding the infrastructure for a telehealth (such as the Extension for Community Healthcare Outcomes project) surveillance system to monitor progress across care cascade and outcomes. In addition, innovative financing mechanisms could be explored to raise money needed to scale up HCV screening and treatment in Pakistan.

The benefit of HCV treatment would also extend beyond those related to slowing progression of liver disease in individuals with HCV infection; successful testing and treatment would be key in the primary prevention of HCV infection by stemming the ongoing risk of primary infections and additional infections. Reducing the incidence of HCV infection may be key to achieving HCV elimination in Pakistan.

We chose Pakistan for this analysis because it has a high prevalence of HCV infection and a large number of infected individuals.^30^ Our results have direct implications for other countries with a high burden of HCV infection. We believe that the WHO targets will not be met unless there is a strong political will and focused effort at both the public and the private levels. Other countries have successfully implemented large-scale initiatives to screen and identify infected individuals. For instance, the Ministry of Health and Population of Egypt set the target to screen 15 million people in 2018.^31^ Elimination of HCV in Pakistan would also need to take precedent among the many competing social issues facing low-income or middle-income nations to prevent millions of deaths due to an otherwise curable disease.

### Limitations

Our study has some limitations. We do not explicitly differentiate between subsequent infection and new infection. Reliable estimates of the incidence of HCV infection in Pakistan are lacking; therefore, we assumed the new incidence to be proportional to the prevalence. We did not include the role of infection prevention interventions in reducing the incidence of HCV infection. Implementing such programs, along with education of practitioners and payers, may be associated with faster reduction in the incidence of HCV infection; thus, our study could have overestimated the incidence of HCV infection. We did not account for the competing risk of mortality from cardiovascular diseases and other infections in patients with HCV infection, which could have resulted in overestimation of the benefits of HCV treatment. However, we also did not account for extrahepatic benefits of HCV treatment, which could have resulted in underestimation of the benefits of HCV treatment. We did not account for the cost associated with strengthening the infrastructure to provide mass screening in Pakistan; therefore, our study could have overestimated cost savings associated with HCV elimination. We accounted for only health care–associated costs and excluded societal costs; inclusion of those costs would have increased the magnitude of cost savings.

## Conclusions

Our study provides insights on the feasibility and cost of HCV elimination in Pakistan. The findings suggest that by substantially scaling up diagnosis and treatment, HCV infection can be eliminated in Pakistan, which may be associated with 323 000 deaths and 13 million HCV-associated DALYs averted from 2018 to 2030. Programs on HCV elimination will require initial investment, but such upfront investment may lead to cost savings in the near future.

## Notes

Supplement. **eAppendix.** Estimation of HCV screening cost **eFigure 1.** Schematic showing the key components and outcomes of HEP-SIM model **eFigure 2.** State-transition model of the natural history of HCV **eFigure 3.** Age distribution for liver-related deaths, 2015-2030 **eTable 1.** Population characteristics of HCV-infected patients in Pakistan **eTable 2.** Pakistan HCV cases age distribution based on national survey and applied to Pakistani age distribution **eTable 3.** Annual transition probabilities for different Markov states used in HEP-SIM model **eTable 4.** SVR rates by treatment, genotype, treatment history, and fibrosis states **eTable 5.** HCV annual uptake of hepatitis C treatment in Pakistan **eTable 6.** Cost of hepatitis C treatment, testing and disease management **eTable 7.** Model outcomes under different scenarios **eReferences**
