# Supplementary material for: Assessment of the Feasibility and Cost of Hepatitis C Elimination in Pakistan
Source: JAMA Netw Open. 2019 May 10;2(5):e193613. doi: 10.1001/jamanetworkopen.2019.3613 (PMC6512462; doi:10.1001/jamanetworkopen.2019.3613)
Supplement: Supplement. — eAppendix. Estimation of HCV screening cost eFigure 1. Schematic showing the key components and outcomes of HEP-SIM model eFigure 2. State-transition model of the natural history of HCV eFigure 3. Age distribution for liver-related deaths, 2015-2030 eTable 1. Population characteristics of HCV-infected patients in Pakistan eTable 2. Pakistan HCV cases age distribution based on national survey and applied to Pakistani age distribution eTable 3. Annual transition probabilities for different Markov states used in HEP-SIM model eTable 4. SVR rates by treatment, genotype, treatment history, and fibrosis states eTable 5. HCV annual uptake of hepatitis C treatment in Pakistan eTable 6. Cost of hepatitis C treatment, testing and disease management eTable 7. Model outcomes under different scenarios eReferences [file jamanetwopen-2-e193613-s001.pdf]

Supplementary Online Content

Chhatwal J, Chen Q, Wang X, et al. Assessment of the feasibility and cost of hepatitis C elimination in Pakistan. *JAMA Netw Open*. 2019;2(5):e193613. doi:10.1001/jamanetworkopen.2019.3613

eAppendix. Estimation of HCV screening cost

eFigure 1. Schematic showing the key components and outcomes of HEP-SIM model

eFigure 2. State-transition model of the natural history of HCV

eFigure 3. Age distribution for liver-related deaths, 2015-2030

eTable 1. Population characteristics of HCV-infected patients in Pakistan

eTable 2. Pakistan HCV cases age distribution based on national survey and applied to Pakistani age distribution

eTable 3. Annual transition probabilities for different Markov states used in HEP-SIM model

eTable 4. SVR rates by treatment, genotype, treatment history, and fibrosis states

eTable 5. HCV annual uptake of hepatitis C treatment in Pakistan

eTable 6. Cost of hepatitis C treatment, testing and disease management

eTable 7. Model outcomes under different scenarios

eReferences

This supplementary material has been provided by the authors to give readers additional information about their work.

eAppendix. Estimation of HCV screening cost

We considered two different types of tests for HCV diagnosis: screening test and test for detection of viremia (**Table 1**). We estimated the cost per HCV case diagnosed separately for *diagnosis via usual care*, and *diagnosis via universal screening*.

For the diagnosis via usual care, we used the cost of the screening test and the test for viremia; and for the diagnosis via universal screening, the average cost per case diagnosed, c_{dx}, was determined by the following approach:

- First we estimated the number of people needed to screen, n_{dx}, to diagnose one chronic HCV-infected patient:

$$n_{dx} = \frac{1}{prev \times p_{chronic} \times sens_{screening} \times sens_{viremia} + (1 - prev \times p_{chronic}) \times (1 - spec_{screening}) \times (1 - spec_{viremia})}$$

- Then we estimated the cost of diagnosing one HCV case, c_{dx} as:

$$c_{dx} = n_{dx} * (c_{screening} + \big((prev \times sens_{screening} + (1 - prev) \times (1 - spec_{screening})\big) \cdot c_{viremia})$$

Where $prev$ represents the seroprevalence of HCV, $p_{chronic}$ represents the chronic rate (proportion of people with HCV+ antibody who have chronic HCV), $c_{screening}$ and $c_{viremia}$ represent the costs of screening test and the test for viremia, respectively, $sens_{screening}, spec_{screening}, sens_{viremia}, spec_{viremia}$ represent the sensitivity and specificity for screening and viremia test, respectively. The estimated values of $c_{screening}$ and $c_{viremia}$ depend on the choice of specific tests.

Performance characteristics of each test are defined below.[2,3]

- Point-of-care screening: 99.5% sensitivity and 99.8% specificity
- Nucleic acid test: 99.8% sensitivity and 99.7% specificity
- GeneXpert: 99.8% sensitivity and 99.7% specificity
- HCVcAg: 93.2% sensitivity and 98% specificity
- HCVcAg + Nucleic acid test if HCVcAg is negative: 99.8% sensitivity and 99.8% specificity

© 2019 Chhatwal J et al. *JAMA Network Open.*

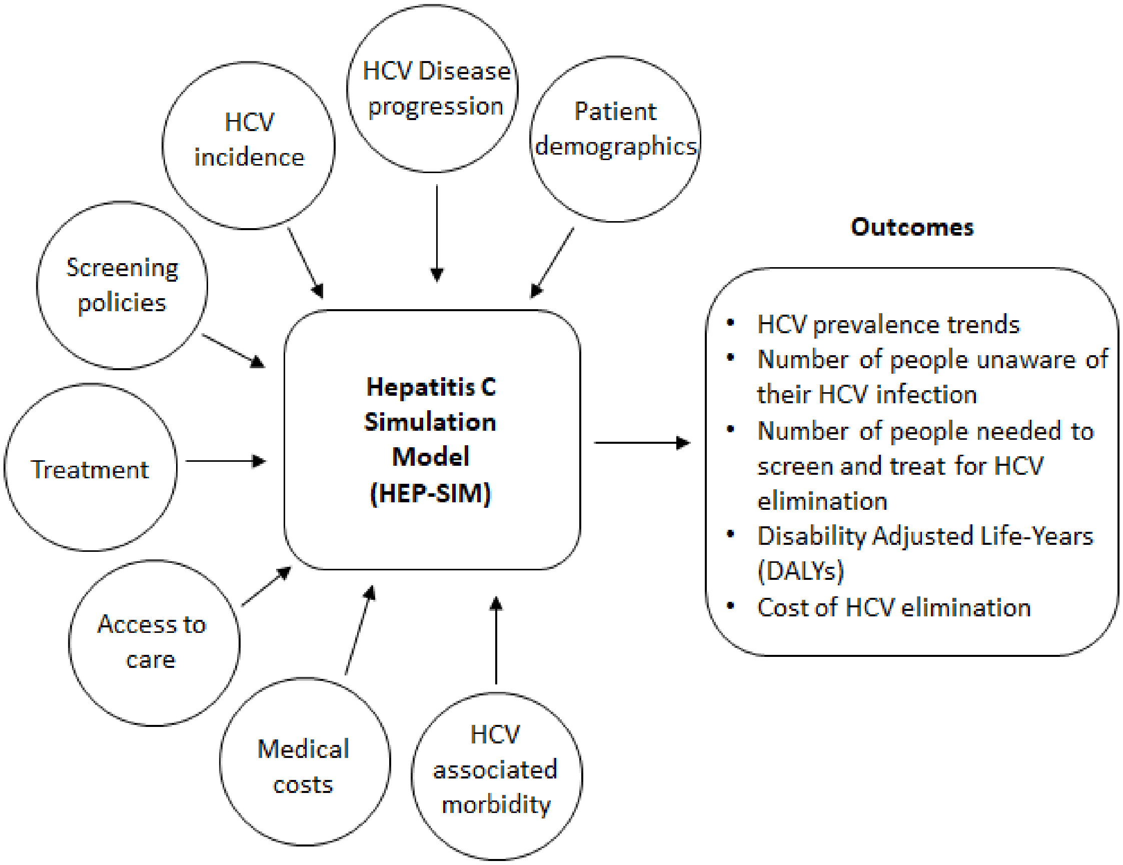

eFigure 1. Schematic showing the key components and outcomes of HEP-SIM model.
HEP-SIM model included patient demographics, HCV disease progression, HCV screening, therapeutic advancement, access to healthcare including insurance status, and the cost of care and treatment. Outcomes of HEP-SIM include temporal trends in HCV prevalence, awareness rate of HCV-infection, HCV-associated advanced sequelae, and budget impact of different interventions.

© 2019 Chhatwal J et al. *JAMA Network Open.*

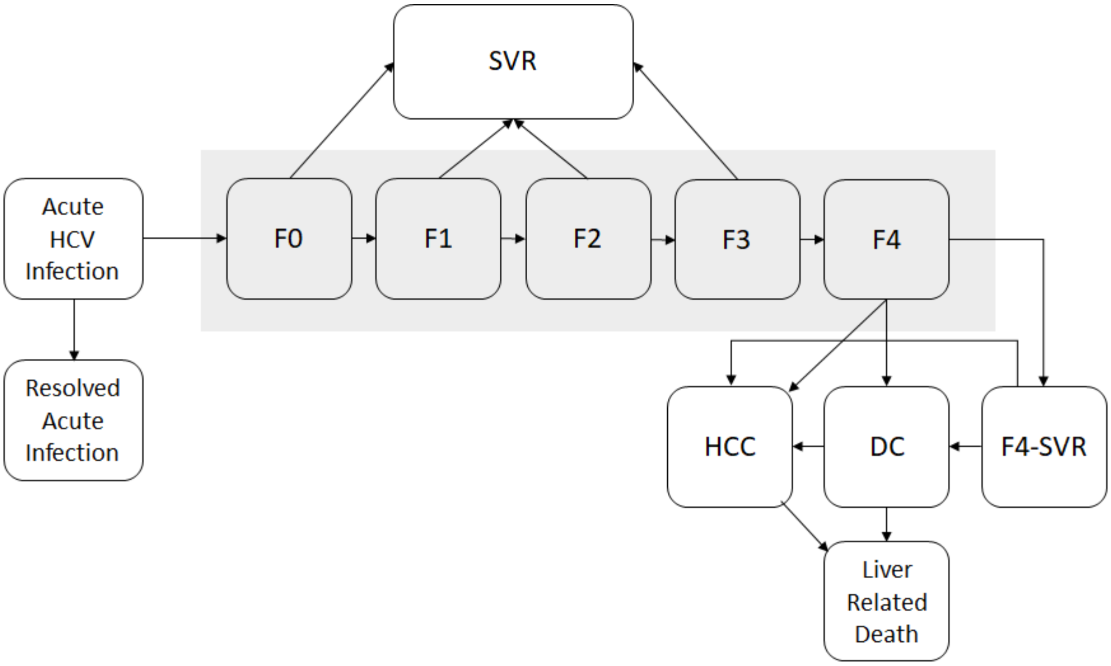

eFigure 2. State-transition model of the natural history of HCV (adapted from Kabiri et al.[1])
At any given time, a patient is represented by one of the health states, which are shown by squares. Arrows between states represent possible transitions based on annual probabilities. Patients whose disease is successfully treated transition to the SVR state. Patients who achieve SVR from F0 to F3 states are assumed to be cured; however, patients in an F4 state who are successfully treated transition to an F4-SVR state and may develop further complications. Patients in HCC, DC, and LT states have a higher mortality rate than the general population. All other patients have the same risk for death as the general population. The probability of death from other causes exists in every state, but deaths from other causes are not shown. According to the Meta-analysis of Histologic Data in Viral Hepatitis (METAVIR) scoring system, F0 indicates no fibrosis of the liver, F1 indicates portal fibrosis without septa, F2 indicates portal fibrosis with few septa, F3 indicates many septa without cirrhosis, and F4 indicates cirrhosis.

Abbreviations: DC, decompensated cirrhosis; HCC, hepatocellular carcinoma; HCV, hepatitis C virus; LT, liver transplantation; SVR, sustained virologic response

© 2019 Chhatwal J et al. *JAMA Network Open.*

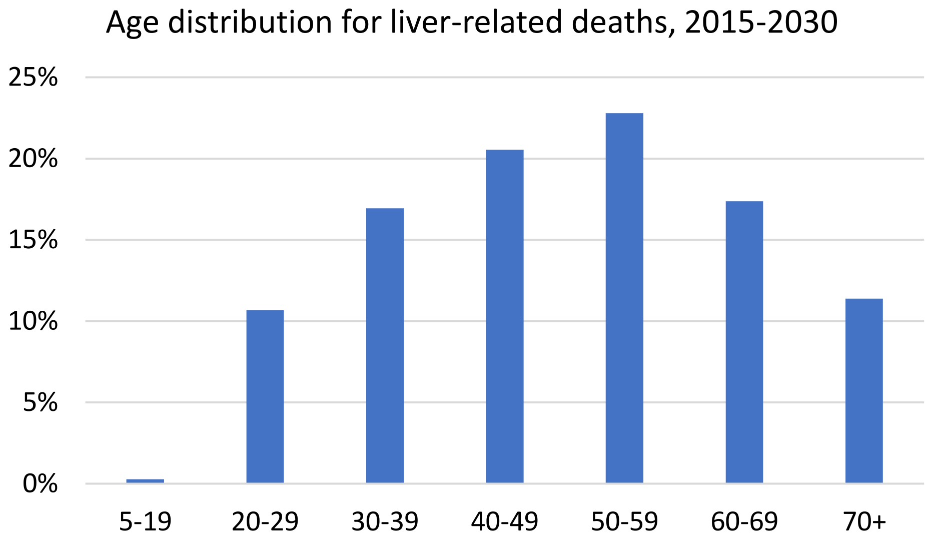

eFigure 3. Age distribution of HCV-related deaths from 2015–2030. From 2015 to 2030, a total of 1.44 million people are projected to die from HCV in Pakistan; 48% of HCV-related deaths would occur in people younger than 50.

© 2019 Chhatwal J et al. *JAMA Network Open.*

eTable 1. Population Characteristics of HCV-Infected Patients in Pakistan

HCV-infected population characteristics	Value	Reference
Total chronic HCV-infected population in 2008 (million)	8.3	4
Chronic infection ratio (%)	74.1%	5
Contraindicated for treatment (%)[a]	34.6	6
Sex (%)		4
Male	52.48	
Female	47.52	
HCV genotype (%)		7
1	11.51	
2	8.41	
3	67.46	
Other	12.62	
Stage distribution of HCV-infected population in 1995[b] (%)		8
F0	27.20	
F1	33.39	
F2	17.11	
F3	11.08	
F4	9.61	
DC	1.43	
HCC	0.18	
Age distribution for HCV-infected population in 2008 (%)		4
0-4	4.7	
5-19	15.3	
20-29	16.4	
30-39	19.7	
40-49	18.6	
50-59	12.3	
60+	12.9	
Proportion of treatment-experienced patients in 1995 (%)	0	HEP-SIM

[a] The ratio of patients with contraindication (with modifiable and non-modifiable reasons) among chronically-infected patients.
[b] We started the model in year 1995 onwards and calibrated model-predicted HCV prevalence in 2008 to data from a national survey in Pakistan.
F0 = no fibrosis; F1 = portal fibrosis without septa; F2 = portal fibrosis with few septa; F3 = numerous septa without cirrhosis; F4 = cirrhosis.

© 2019 Chhatwal J et al. *JAMA Network Open.*

eTable 2 Pakistan HCV cases age distribution based on national survey and applied to Pakistani age distribution. [4]

Pakistan age distribution	Population by age 2008	HCV prevalence (%)	HCV cases	HCV case age distribution (%)
**0-4**	20,430,456	1.9	388,179	4.74
**5-9**	20,133,813	2.1	422,810	5.16
**10-14**	20,362,502	2.1	427,613	5.22
**15-19**	19,088,301	2.1	400,854	4.89
**20-24**	16,833,804	4.4	740,687	9.04
**25-29**	13,685,642	4.4	602,168	7.35
**30-34**	11,283,919	7.8	880,146	10.74
**35-39**	9,454,026	7.8	737,414	9.00
**40-44**	8,296,061	9.9	821,310	10.02
**45-49**	7,104,877	9.9	703,383	8.58
**50-54**	5,548,910	10.4	577,087	7.04
**55-59**	4,182,016	10.4	434,930	5.31
**60-64**	3,480,358	10.0	348,036	4.25
**65-69**	2,851,939	10.0	285,194	3.48
**70-74**	1,985,374	10.0	198,537	2.42
**75-79**	1,261,872	10.0	126,187	1.54
**80-84**	667,228	10.0	66,723	0.81
**85+**	356,985	10.0	35,699	0.44
**Total**	167,008,083		8,196,956	100%

Because the above survey excluded people who injected drugs, [4] we added HCV cases among people using drugs based on data from Pakistan AIDS Control Program that estimated that in 2015, 104,804 people injected drugs in Pakistan and 89% of people who inject drugs had chronic HCV.[9,10]

© 2019 Chhatwal J et al. *JAMA Network Open.*

eTable 3. Annual transition probabilities for different Markov states used in HEP-SIM model

Input	Base case	Values for sensitivity analysis			
		**Range**	**Distribution**	**Alpha**	**Beta**
**Transition *probabilities (annual)***					
F0 to F1 [11]	0.117	0.104–0.130	Beta	274.98	2,075.30
F1 to F2 [11]	0.085	0.075–0.096	Beta	210.06	2,261.18
F2 to F3 [11]	0.120	0.109–0.133	Beta	288.05	2,112.38
F3 to F4 [11]	0.116	0.104–0.129	Beta	270.61	2,062.22
F4 to DC [12]	0.039	0.010–0.079	Beta	3.51	86.48
F4 to HCC [12]	0.014	0.010–0.079	Beta	0.18	12.38
Post F4-SVR to DC [13]	0.008	0.002–0.036	Beta	0.31	38.58
Post F4-SVR to HCC [13]	0.005	0.002–0.013	Beta	1.49	297.13
DC to HCC [14]	0.068	0.030–0.083	Beta	73.58	1008.49
DC (first year) to death from liver disease [14]	0.182	0.065–0.190	Beta	1626.40	7309.88
DC (subsequent year) to death from liver disease [14]	0.112	0.065–0.190	Beta	7.03	55.77
HCC to death from liver disease [12]	0.427	0.330–0.860	Beta	2.14	2.87

Abbreviations: SVR, sustained virologic response; F0–F4, METAVIR fibrosis score; DC, decompensated cirrhosis; HCC, hepatocellular carcinoma; F4-SVR. Post-SVR state of treated cirrhotic patient

© 2019 Chhatwal J et al. *JAMA Network Open.*

eTable 4. SVR rates by treatment, genotype, treatment history, and fibrosis states

Treatment history and fibrosis state	GT1	GT2	GT3	GT4–6	References
**PEG+RBV**					
Treatment naïve					1,15-19
F0-F3	0.54	0.82	0.70	0.58	
F4	0.36	0.64	0.49	0.32	
Contraindicated with modifiable reasons					
F0-F2	-	0.66	0.56	0.46	
F3	0.43	0.66	0.56	0.46	
F4	0.28	0.51	0.40	0.26	
Failed PEG+RBV: relapse					
F0-F3	0.27	0.71	0.66	0.31	
F4	0.13	0.56	0.52	0.24	
Failed PEG+RBV: partial response					
F0-F3	0.18	0.69	0.64	0.31	
F4	0.10	0.55	0.51	0.24	
Failed PEG+RBV: null response					
F0-F3	0.10	0.54	0.50	0.31	
F4	0.05	0.42	0.39	0.24	
**DAA NS5A[1]**					
Treatment naïve, contraindicated, failed PEG+RBV, failed BOC/TEL+PEG+RBV (GT1 only), failed DAA non-NS5A, failed DAA nonNS5A					20-28
F0-F3	0.95	0.99	0.95	0.99	
F4	0.9	0.99	0.9	0.99	

[1]DAA1 NS5A includes any of the following drug combinations: LDV/SOF+/-RBV, SOF+DCV, DCV+PEG+/-RBV, and SOF/VEL.

Abbreviations: GT, genotype; PEG, peginterferon; RBV, ribavirin; BOC, boceprevir; TEL, telaprevir; DAA, direct-acting antiviral; NS5A, nonstructural protein 5A; SOF, sofosbuvir; LDV, ledipasvir; DCV, daclatasvir; VEL, velpatasvir.

© 2019 Chhatwal J et al. *JAMA Network Open.*

eTable 5. HCV Annual Uptake of Hepatitis C Treatment in Pakistan

Year	Treated Cases
**2004**	11,809
**2005**	19,828
**2006**	30,675
**2007**	50,857
**2008**	70,499
**2009**	85,000
**2010**	85,000
**2011**	85,000
**2012**	85,000
**2013**	85,000
**2014**	85,000
**2015**	65,385
**2016**	160,650
**2017**	160,650

Source: Polaris observatory data (http://cdafound.org/polaris-hepC-dashboard/, extracted in October 2017)

© 2019 Chhatwal J et al. *JAMA Network Open.*

eTable 6. Cost of hepatitis C treatment, testing and disease management

Cost	Value ($)
Health state costs (annual)[a]	
F0	27
F1	27
F2	28
F3	56
Compensated cirrhosis	63
Decompensated Cirrhosis	636
Hepatocellular Cancer	1216
**Testing cost (one-time)**	
Laboratory-based antibody test	18
Point-of-care antibody test (screening)	5
GeneXpert	20
HCVcAg test	25
NAT	137
**Treatment cost (one-time)**	
Peginterferon-ribavirin	60
DAA	60

Abbreviations: SVR, sustained virologic response; F0–F4, METAVIR fibrosis score; DC, decompensated cirrhosis; HCC, hepatocellular carcinoma; F4-SVR. Post-SVR state of treated cirrhotic patient ; NAT, nucleic acid test ; DAA, direct-acting antiviral; HCVcAg, HCV core antigen.

[a]We estimated annual healthcare costs associate HCV disease management using WHO CHOICE tool. In particular, we extracted inpatient and outpatient primary costs from the World Health Organization's CHOosing Interventions that are Cost Effective (CHOICE) project (https://www.who.int/choice/toolkit/en/) and took the weighted average of cost per inpatient visit and cost per outpatient visit for each HCV-associated health state. The ratios of weights (inpatient: outpatient) were 0.38:0.62 for F0–F4, 0.43:0.57 for compensated cirrhosis, 0.66:0.34 for decompensated cirrhosis, and 0.55:0.45 for hepatocellular carcinoma as reported by McAdam Marx.[7] We then estimated the ratio of the above costs in Pakistan to United States. Finally, we estimated Pakistan specific HCV disease costs by multiplying this ratio with HCV costs in the United States as reported in McAdam Marx et al.[29] and Chhatwal et al.[6]

© 2019 Chhatwal J et al. *JAMA Network Open.*

eTable 7 Model outcomes under different scenarios

Key model outcomes	Base case	Increasing HCV incidence by 2% per year	HCV prevalence increased by 20% in 2018[a]	HCV awareness rate of 7% in 2018[b]
**To reach HCV elimination**				
**Number diagnosed per year**	900,000	950,000	1,050,000	1,050,000
**Number treated per year**	700,000	800,000	900,000	700,000
**HCV prevalence in 2030**	89,191	222,430	82,697	103,466
**Reduction compared to 2015**	98.9%	97.3%	99.2%	98.8%
**HCV incidence in 2030**	9,181	364,000	4,616	12,908
**Reduction compared to 2015**	96.7%	-- [c]	98.6%	95.4%
**Number of death averted between 2015–2030**	323,443	329,952	381,409	391,003
**DALYs averted between 2015-2030**	13,011,508	13,252,919	15,325,227	15,658,989
**Total cost of HCV management between 2018-2030 ($, millions)**	10,006	11,199	11,652	10,410
**Cost-saving from new diagnostic tests between 2018-2030 ($, millions)**	2,551	2,945	3,167	2,961

[a] The current HCV prevalence is 20% higher (i.e., 9.6 million in 2018) than that in the base case (with 8.0 million).

[b] The current HCV awareness rate is 7% (in contrast to 12.7% in base case in 2018).

[c] For the "Increasing HCV Incidence" Scenario, the incidence reduction target cannot be achieved because the increasing incidence was used as model inputs. The diagnosis and treatment rates were selected based on the feasibility of other three elimination targets.

© 2019 Chhatwal J et al. *JAMA Network Open.*

eReferences

1. Kabiri M, Jazwinski AB, Roberts MS, Schaefer AJ, Chhatwal J. The changing burden of hepatitis C in the United States: Model-based predictions. *Annals of internal medicine* 2014; **161**(3): 170-80.
2. Fourati S, Feld JJ, Chevaliez S, Luhmann N. Approaches for simplified HCV diagnostic algorithms. 2018; **21**(S2): e25058.
3. Chevaliez S, Pawlotsky J-M. New virological tools for screening, diagnosis and monitoring of hepatitis B and C in resource-limited settings. *Journal of hepatology* 2018; **69**(4): 916-26.
4. Qureshi H, Bile KM, Jooma R, Alam SE, Afridi HU. Prevalence of hepatitis B and C viral infections in Pakistan: findings of a national survey appealing for effective prevention and control measures. *Eastern Mediterranean health journal = La revue de sante de la Mediterranee orientale = al-Majallah al-sihhiyah li-sharq al-mutawassit* 2010; **16 Suppl**: S15-23.
5. Ahmed W, Qureshi H, Arif A, Alam SE. Changing trend of viral hepatitis--"A twenty one year report from Pakistan Medical Research Council Research Centre, Jinnah Postgraduate Medical Centre, Karachi". *JPMA The Journal of the Pakistan Medical Association* 2010; **60**(2): 86-9.
6. Chhatwal J, Chen Q, Aggarwal R. Estimation of Hepatitis C Disease Burden and Budget Impact of Treatment Using Health Economic Modeling. *Infectious Disease Clinics* 2018; **32**(2): 461-80.
7. Idrees M, Riazuddin S. Frequency distribution of hepatitis C virus genotypes in different geographical regions of Pakistan and their possible routes of transmission. *BMC infectious diseases* 2008; **8**: 69.
8. Ahmad W, Ijaz B, Javed FT, et al. A comparison of four fibrosis indexes in chronic HCV: development of new fibrosis-cirrhosis index (FCI). *BMC gastroenterology* 2011; **11**: 44.
9. Bergenstrom A, Achakzai B, Furqan S, ul Haq M, Khan R, Saba M. Drug-related HIV epidemic in Pakistan: a review of current situation and response and the way forward beyond 2015. *Harm Reduct J* 2015; **12**: 43.
10. Mumtaz GR, Weiss HA, Thomas SL, et al. HIV among people who inject drugs in the Middle East and North Africa: systematic review and data synthesis. *PLoS medicine* 2014; **11**(6): e1001663.
11. Thein H, Yi Q, Dore G, Krahn M. Estimation of stage specific fibrosis progression rates in chronic hepatitis C virus infection: A meta analysis and meta regression. *Hepatology (Baltimore, Md)* 2008; **48**(2): 418-31.
12. Fattovich G, Giustina G, Degos F, et al. Morbidity and mortality in compensated cirrhosis type C: a retrospective follow-up study of 384 patients. *Gastroenterology* 1997; **112**(2): 463-72.
13. Cardoso AC, Moucari R, Figueiredo-Mendes C, et al. Impact of peginterferon and ribavirin therapy on hepatocellular carcinoma: incidence and survival in hepatitis C patients with advanced fibrosis. *Journal of hepatology* 2010; **52**(5): 652-7.
14. Planas R, Ballesté B, Antonio Álvarez M, et al. Natural history of decompensated hepatitis C virus-related cirrhosis. A study of 200 patients. *Journal of hepatology* 2004; **40**(5): 823-30.
15. Shiffman ML, Suter F, Bacon BR, et al. Peginterferon Alfa-2a and Ribavirin for 16 or 24 Weeks in HCV Genotype 2 or 3. *New England Journal of Medicine* 2007; **357**(2): 124-34.
16. McHutchison JG, Lawitz EJ, Shiffman ML, et al. Peginterferon Alfa-2b or Alfa-2a with Ribavirin for Treatment of Hepatitis C Infection. *New England Journal of Medicine* 2009; **361**(6): 580-93.
17. Talal A, LaFleur J, Hoop R, et al. Absolute and relative contraindications to pegylated-interferon or ribavirin in the US general patient population with chronic hepatitis C: results from a US database of over 45 000 HCV-infected, evaluated patients. *Alimentary pharmacology & therapeutics* 2013; **37**(4): 473-81.
18. Shiffman ML, Di Bisceglie AM, Lindsay KL, et al. Peginterferon Alfa-2a and ribavirin in patients with chronic hepatitis C who have failed prior treatment1, 2, 3 1 2 3. *Gastroenterology* 2004; **126**(4): 1015-23.
19. Poynard T, Colombo M, Bruix J, et al. Peginterferon alfa-2b and ribavirin: effective in patients with hepatitis C who failed interferon alfa/ribavirin therapy. *Gastroenterology* 2009; **136**(5): 1618-28.
20. Kowdley KV, Gordon SC, Reddy KR, et al. Ledipasvir and sofosbuvir for 8 or 12 weeks for chronic HCV without cirrhosis. *New England Journal of Medicine* 2014; **370**(20): 1879-88.
21. Sulkowski MS, Gardiner DF, Rodriguez-Torres M, et al. Daclatasvir plus sofosbuvir for previously treated or untreated chronic HCV infection. *New England Journal of Medicine* 2014; **370**(3): 211-21.
22. Afdhal N, Reddy KR, Nelson DR, et al. Ledipasvir and sofosbuvir for previously treated HCV genotype 1 infection. *The New England journal of medicine* 2014; **370**(16): 1483-93.
23. Feld JJ, Kowdley KV, Coakley E, et al. Treatment of HCV with ABT-450/r-ombitasvir and dasabuvir with ribavirin. *The New England journal of medicine* 2014; **370**(17): 1594-603.

© 2019 Chhatwal J et al. *JAMA Network Open.*

24. Ferenci P, Bernstein D, Lalezari J, et al. ABT-450/r–Ombitasvir and Dasabuvir with or without Ribavirin for HCV. *New England Journal of Medicine* 2014; **370**(21): 1983-92.
25. Poordad F, Hezode C, Trinh R, et al. ABT-450/r-ombitasvir and dasabuvir with ribavirin for hepatitis C with cirrhosis. *The New England journal of medicine* 2014; **370**(21): 1973-82.
26. Zeuzem S, Jacobson IM, Baykal T, et al. Retreatment of HCV with ABT-450/r-ombitasvir and dasabuvir with ribavirin. *The New England journal of medicine* 2014; **370**(17): 1604-14.
27. Flisiak R, Janczewska E, Wawrzynowicz-Syczewska M, et al. Real-world effectiveness and safety of ombitasvir/paritaprevir/ritonavir +/- dasabuvir +/- ribavirin in hepatitis C: AMBER study. *Alimentary pharmacology & therapeutics* 2016; **44**(9): 946-56.
28. Feld JJ, Jacobson IM, Hézode C, et al. Sofosbuvir and Velpatasvir for HCV Genotype 1, 2, 4, 5, and 6 Infection. *New England Journal of Medicine* 2015; **373**(27): 2599-607.
29. McAdam-Marx C, McGarry LJ, Hane CA, Biskupiak J, Deniz B, Brixner DI. All-cause and incremental per patient per year cost associated with chronic hepatitis C virus and associated liver complications in the United States: A managed care perspective. *J Manag Care Pharm* 2011; **17**(7): 531-46.

© 2019 Chhatwal J et al. *JAMA Network Open.*
